# Exploring computerised cognitive training as a therapeutic intervention for people with Huntington’s disease (CogTrainHD): protocol for a randomised feasibility study

**DOI:** 10.1186/s40814-018-0237-0

**Published:** 2018-02-06

**Authors:** Emma Yhnell, Hannah Furby, Rachel S. Breen, Lucy C. Brookes-Howell, Cheney J. G. Drew, Rebecca Playle, Gareth Watson, Claudia Metzler-Baddeley, Anne E. Rosser, Monica E. Busse

**Affiliations:** 1 Neuroscience and Mental Health Research Institute, Cardiff University, 3rd Floor, Hadyn Ellis Building, Maindy Road, Cardiff, CF24 4HQ Wales UK; 2 Cardiff University Brain Research Imaging Centre (CUBRIC), School of Psychology, Cardiff University, Maindy Road, Cardiff, CF24 4HQ Wales UK; 3 Centre for Trials Research (CTR), Cardiff University, 4th and 7th Floors, Neuadd Meironnydd, Heath Park, Cardiff, CF14 4YS Wales UK

**Keywords:** Huntington’s disease, Cognition, Computerised cognitive training, Feasibility study

## Abstract

**Background:**

Cognitive impairments, especially deficits of executive function, have been well documented as a core and early feature in Huntington’s disease (HD). Cognitive impairments represent considerable burden and can be devastating for people and families affected by HD. Computerised cognitive training interventions that focus on improving executive function present a possible non-pharmacological treatment option. We propose to determine the feasibility, acceptability, and appropriate outcome measures for use in a randomised controlled feasibility study.

**Methods/design:**

Participants will be randomised into either a computerised cognitive training group or a control group. Those randomised to the training group will be asked to complete a cognitive training intervention based on the HappyNeuron Pro software tasks of executive function, for a minimum of 30 min, three times a week for the 12-week study duration. Participants in the control group will not receive computerised cognitive training but will receive a similar degree of social interaction via equivalent study and home visits. We will explore quantitative outcome measures, including measures of cognitive performance, motor function, questionnaires and semi-structured interviews, as well as magnetic resonance imaging (MRI) measures in a subset of participants. Feasibility will be determined through assessment of recruitment, retention, adherence and acceptability of the intervention.

**Discussion:**

The results of this study will provide crucial guidance and information regarding the feasibility of conducting a randomised controlled study into computerised cognitive training in HD. This study is crucial for the development of larger definitive randomised controlled trials which are powered to determine efficacy and for the development of future cognitive training programmes for people affected by HD.

**Trial registration:**

The study is registered on clinicaltrials.gov and has the unique identifier NCT02990676.

## Background

### Background and rationale

Huntington’s disease (HD) is caused by a CAG repeat trinucleotide expansion within the first exon of the *huntingtin* gene [1], and it causes a range of symptoms including motor, cognitive and psychiatric disturbances [2–8]. These disease symptoms significantly affect daily activities, independence and quality of life, even during the early stages of the disease [9, 10]. Cognitive dysfunctions early in the HD disease process have been well documented and can include specific problems with attention, cognitive flexibility and memory [11, 12]. Therefore, cognitive training interventions, which focus on tasks that require executive function, present a potentially exciting non-pharmacological treatment option for neurodegenerative diseases including HD. Studies in HD mice have previously demonstrated executive function impairments [13, 14], and subsequent studies have shown that cognitive training can benefit HD mice and modify the associated cognitive and motor disease symptoms [15, 16]. These results provide important ‘proof of principle’ evidence that an early cognitive training intervention may be beneficial in HD.

Furthermore, there is accumulating evidence in both healthy and clinical populations that cognitive training, via repeatedly conducting tasks that require specific aspects of executive function such as attention, reasoning, dual tasking and memory, can improve cognitive functions in the trained domain [17–20]. For instance, computerised cognitive training on such tasks has been found to improve cognitive impairments in Alzheimer’s disease [21–23] and Parkinson’s disease [24–26]. In addition, a recent single arm feasibility study demonstrated the feasibility of and adherence to working memory training on the Cogmed QM programme in a small sample of HD patients [27]. Furthermore, previous studies have found that training-related changes are associated with alterations in white matter, particularly with regard to altered myelination in both healthy controls [28] and HD [20]. Thus, we intend to use MRI measures to explore the potential neural mechanisms which may be associated with computerised cognitive training in the HD clinical population.

The utilisation of computerised cognitive training strategies may provide several advantages over repeating practical tasks [17, 18]. Computerised cognitive training strategies are automated and often provide adaptive difficulty levels based on participant performance. Furthermore, they can be completed by participants at their convenience, they provide automated tasks which require comparatively little demand on motor function and they provide several objective outcome measures which can be used to detect subtle changes in function [21–26]. Furthermore, the HappyNeuron cognitive training software, that we intend to use in this study, can be made available in different languages to allow for consistent training globally, and it provides an interface for the researcher to observe the progress of participants.

Despite this, computerised cognitive training interventions are yet to be extensively investigated in the HD clinical population. Therefore, this feasibility study will clarify key uncertainties regarding the study design, participant eligibility and willingness of potential participants to consent to the study and be randomised as well as recruitment, retention and acceptability. It will also facilitate the evaluation of outcome measures and an estimation of effect of the intervention. This study is a necessary step before proceeding to a fully powered efficacy trial in the HD patient population [29].

### Aims and objectives

The primary aim of this study is to determine if computerised cognitive training is feasible and acceptable for people with HD in the setting of a randomised controlled study. A secondary aim is the exploration of outcome measures of cognitive function, motor function and questionnaires. Furthermore, we will explore possible mechanistic measures of training-related plasticity through magnetic resonance imaging (MRI) metrics.

#### Primary objective

(i) To assess the feasibility of delivering a home-based computerised cognitive training programme for people with HD, utilising both quantitative and qualitative measures considering:

- The willingness of eligible participants to receive the intervention and participate in a randomised controlled study
- Potential barriers to recruitment
- Potential barriers to completion of the study
- Response rates and adherence to the computerised cognitive training programme

#### Secondary objectives

(i) To explore group effect estimates between the cognitive training intervention group in comparison to the control group.
(ii) To use MRI techniques in a subset of participants to explore the potential neural mechanisms of brain plasticity that may underpin any observed effects.
(iii) To evaluate the intervention using participant and family member/carer feedback to inform future trials in this patient population.

### Study design

The proposed study is a randomised feasibility study of an online computerised cognitive training intervention for people with HD, to be completed in the home. Those allocated to the control group will be asked to continue as normal. All participants will receive home visits. The home visits will include a battery of cognitive tests for both groups; those allocated to the intervention will receive support in setting up the computerised cognitive training software. Within the overall study, a subgroup of 16 participants will undergo MRI at baseline, and approximately 6 weeks into the intervention, MRI will be offered to participants on a first come first served basis for those who are eligible and willing. The feasibility of repeated imaging in this patient population will be ascertained, and an exploration of the most appropriate MRI metrics will also be undertaken as part of the feasibility study.

**Fig. 1:**
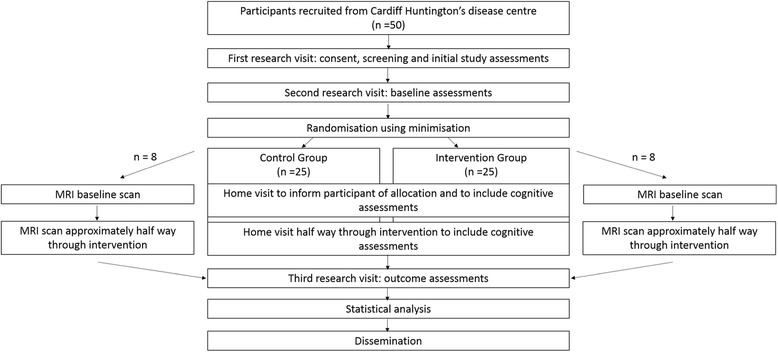
Schematic representation of feasibility study

The study schema is illustrated in Fig. 1.

## Methods

### Participants, interventions and outcomes

#### Study setting

Participants will be recruited from the Cardiff HD Centre at Cardiff University. Study assessments will take place in the Hadyn Ellis Building (HEB) and Cardiff University Brain Research Imaging Centre (CUBRIC). Many patients attending the centre are already participating in the Enroll-HD observational study (REC no. 04/WSE05/89), which longitudinally monitors the progression of disease symptoms. Enroll-HD participants provide permission to be contacted about other HD research studies and for their coded data to be accessed by researchers conducting HD-related research; therefore, these participants will be approached for this study. As such, a full clinical data set including full medical and medication history will be available for each research participant. In addition, we will seek consent to approach participant family members, friends and carers for interview, although involvement of a family member, friend or carer is not a requirement for participation in the study.

#### Eligibility criteria

Inclusion and exclusion criteria for the study are shown in Table 1. The inclusion and exclusion criteria are broad to determine feasibility for a range of disease stages which will inform the design and delivery of future trials.

**Table 1:** Inclusion and exclusion criteria

Inclusion criteria	Exclusion criteria
• Confirmed HD diagnosis by genetic test. • Over 18 years of age. • Enrolled in the Enroll-HD study. • Stable medication regime 4 weeks prior to recruitment (and not anticipated to change medications during the study period).	• Inability to provide consent. • Any known neurological condition (other than HD). • Currently actively involved in any other interventional trial (i.e. have begun the intervention) or within 4 weeks of completing the final assessments of an interventional trial. • Currently regularly completing a computerised cognitive training intervention. • For the subset of participant’s eligible and willing to have an MRI scan, MRI contraindications (e.g. a pacemaker) as established using CUBRIC’s standard screening procedures*.* In addition, significant levels of disease-related chorea will exclude participants from MRI.

#### The cognitive training intervention

The 12-week cognitive training intervention, using HappyNeuron Pro software, will be completed in participant homes. The software is automated, such that it provides non-biased data recording and an interface which allows the researcher to track compliance remotely. It has previously been validated in both healthy controls [30] and patients with depression [31] and has shown patient benefit including improved cognition and functionality.

The cognitive training intervention will comprise six tasks developed by HappyNeuron which are specifically aimed at training within the executive function domain.

These tasks include:

1. ‘Writing in the stars’

The participant is given a list of nine words. Only six of them can be used to fill the empty squares and connect with each other to form the six-point star. This exercise aims to train logical reasoning in order to determine which six words to choose from the list of nine, to find which positions of letters are common to two or three words.

2. ‘Basketball in New York’

The participant sees a first line of three hoops with coloured basketballs inside. In the second line, they will have to determine the number of basketball moves that are required to reach the same configuration as the first line of hoops. This exercise aims to train visual mental imagery as all the possible actions must be visualised in order to complete the task.

3. ‘Decipher’

The participant is asked to decipher quotations where the letters have been replaced, either by letters or by symbols. Each letter is always replaced by the same letter or symbol. This exercise is designed to train the participant’s concentration, language (spelling, grammar rules, letter frequency), logic and the capacity to make deductions from hypotheses.

4. ‘The Towers of Hanoi’

The participant must configure coloured rings on a series of pegs to match a target image. There are a number of rules. The participant can only move one ring at a time and can never put a larger ring on top of a smaller ring. This task is designed to train temporal-spatial design, memory and planning skills. This computerised task is directly related to the ‘real life’ version which will be completed during the participant home visits; thus, we will investigate any possible transfer effects of this training.

5. ‘Hurray for change’

This task comprises two exercises. In the first part, a series of 4 to 16 letters or words must be linked in alphabetical order. The second part demands that the participant alternately sorts two series of items. This exercise requires concentration and visual and spatial exploration skills. It is designed to train mental flexibility, visual and verbal working memory and language skills. This online task is similar in nature to the trail making tasks which are included as part of the written study assessments. Thus, we will analyse any transfer effects.

6. ‘The right count’

In this exercise, the participant is presented with a series of numbers and mathematical symbols to perform mental arithmetic calculations. This game trains working memory, executive function (calculation and arithmetic reasoning) and mental imagery.

The tasks have an adaptive design in that when completing the tasks, participants are required to achieve an 80% level of accuracy twice within the testing period in order to progress to the next level of the task. If a participant achieves less than 60% accuracy on a task, they will be automatically moved to a lower difficulty level. There is a maximum of nine difficulty levels in each task available to participants. If a participant reaches level nine, they will be required to continue at this level for the remainder of the training. Performance data is recorded at the completion of each game and is transmitted and stored on the HappyNeuron server.

Participants will be asked to complete the HappyNeuron Pro training for a minimum of 30 min three times a week for 12 weeks, based on the recommendations of the software provider. Completion of the cognitive training programme will be supported by email or telephone reminders and remotely monitored by the researchers through an interface. The data generated during the intervention, including the tasks completed, duration of tasks and levels achieved will be stored according to HappyNeuron policies and exported to the researchers upon request.

Participants allocated to the control group will be asked to continue as normal for the 12-week duration. The control participants will also receive home visits, to control for increased social interaction.

#### Involvement of nominated carers, friends or family members

The intervention is designed to be completed independently by the participant. However, as part of this feasibility study, we will be investigating if any nominated carers, family members or friends help or aid any of the participants in completing the intervention. Participants and nominated carers, friends or family members will be instructed that they may help the participant if they feel able and willing, although they are not specifically required to do so. However, they must not complete the training for the participant. The involvement of carers, family members or friends may be an important aspect of the study and something that will be specifically explored in the semi-structured interviews in order to inform future trials.

#### Outcome assessments

As this feasibility study is exploratory in nature, the outcome measures selected for inclusion in this study will be utilised to inform future larger trials. Baseline and outcome assessments will be completed during the study (Table 2), in addition to MRI measures which will be completed at baseline and 6 weeks into the intervention. An extended battery of cognitive assessments will be completed during home visits (Table 2).

**Table 2:**
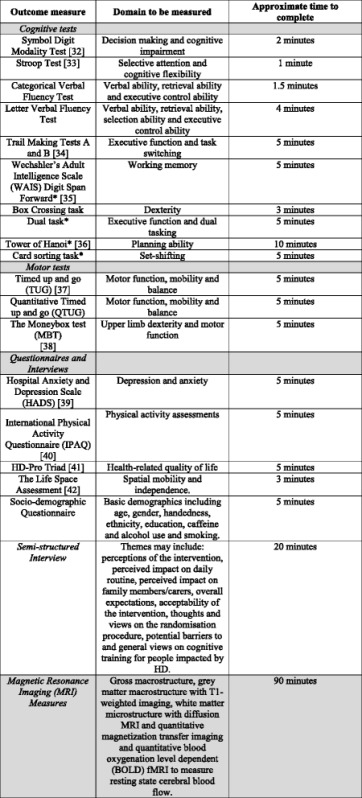
Study outcome assessments

Overall assessment categories are indicated in shaded boxes

*Tests to be included in the extended battery of cognitive assessments to be completed in participant homes during study home visits

#### Participant timeline

The participant timeline is shown in Table 3.

**Table 3:**
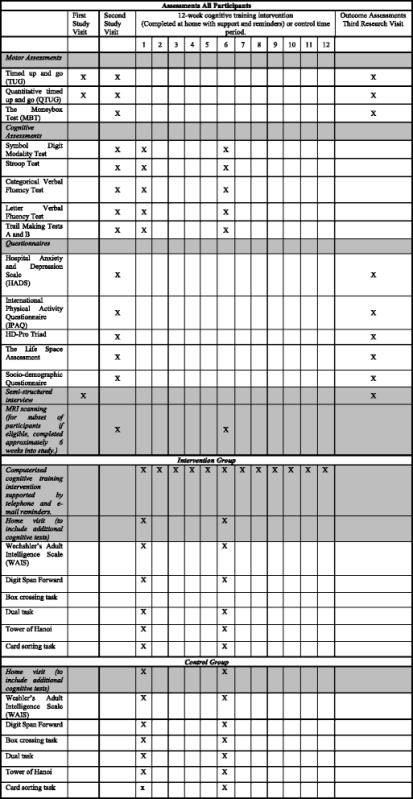
Schema of assessments and intervention

Shaded areas indicate subgroups of study assessments

#### Withdrawal and loss to follow-up

If a participant wishes to withdraw from the study, they can inform the researchers conducting the study or any member of the research team at any time, in person, via telephone or in writing. If a participant initially consents but subsequently withdraws from the study, a clear distinction will be made as to what aspect of the study the participant is withdrawing from utilising a study withdrawal form.

#### Sample size

This is an exploratory feasibility study which aims to establish the feasibility of conducting a randomised controlled study into computerised cognitive training intervention in the HD patient population and provide evidence for a formal power calculation. We will aim to recruit 50 participants with a target of randomising 40 participants. The suggested numbers are based on previous literature regarding cognitive training in other diseases [21–27]. A subgroup of 16 participants (*n* = 8 in the training and *n* = 8 in the control group) will undergo MRI scanning before and 6 weeks into the intervention.

#### Recruitment

An invitation letter and information sheet will be sent to potential participants who are already participating in the Enroll-HD study. In addition, Enroll-HD participants may be approached during their clinical visits and will be informed of the study. Potential participants will be given sufficient time to ask questions, discuss the study with the researchers and discuss the study further with their family and friends. Potential participants who wish to participate will be reminded that they can change their mind or withdraw without reason at any time and that their decision will not affect the standard care that they receive. Willing participants will be asked to sign a consent form, and the initial assessments will then begin.

All potential participants will be screened for their eligibility based on the inclusion/exclusion criteria. MRI scanning is an additional component of the study for those who are eligible. Thus, as part of the screening process, eligible participants will be screened for contraindications to MRI and will be asked to sign an additional consent form which ensures that they understand the potential risks of MRI.

### Assignment of interventions

#### Sequence generation, allocation concealment mechanism and implementation

Randomisation will be performed using a minimisation procedure utilising Minim computer software [32] to ensure balance between the groups for categorical variables of age and cognitive function (determined by the categorical verbal fluency test). Age and cognitive function were chosen as balancing variables, as they have been shown to be significantly associated with cognitive ability [33, 34]; thus, they will be given the same weighting during the minimisation procedure. Allocation and the minimization procedure will be performed by an independent statistician who will input the balancing variables into the Minim software in order to provide the group allocation (intervention or control) for each research participant.

#### Blinding (masking)

Blinding is not used in this study, due to limited resources and staffing. Participants allocated to the intervention group will know that they have been allocated to this group as they are required to complete the cognitive training intervention. The lack of blinding is a limitation of the study design; however, quantitative outcome measures have been utilised where possible in order to minimise the possible bias that may be introduced due to a lack of blinding.

### Data collection, management and analysis

#### Data collection methods

Data will be collected on paper case report forms (CRFs), before subsequent entry into an online database. Semi-structured interviews will be recorded on a Dictaphone before being transcribed verbatim prior to subsequent analysis. MRI data will be stored on the secure CUBRIC database and downloaded on to secure Cardiff University user accounts to enable further processing and statistical analysis.

#### Data management

Electronic data will be stored within firewall- and password-protected computer systems. No data, whether paper or electronic, will leave the Cardiff University site without being completely anonymised. At the conclusion of the study, patient identifiable data will be destroyed and non-identifiable data will be archived for 15 years, in line with Cardiff University policies. Anonymised data may be shared with researchers at Cardiff University and with other research organisations in the UK; it may also be made publicly available for future research use or shared with the organisations who are funding the study. In the case of the data generated using HappyNeuron systems, these data are captured centrally and are therefore subject to HappyNeuron’s policies and procedures. All participant identification and referral procedures as well as procedures for data storage, processing and management will comply with the Data Protection Act 1998. Data collected on paper forms will be stored in locked cabinets in a swipe card access-controlled building, in compliance with the guidelines set by the Cardiff University Research Governance Framework.

#### Statistical methods

Adherence to the proposed cognitive training intervention and any serious adverse events (SAEs) will be reported alongside participant withdrawals. Summary statistics of demographics will be reported for both the control and intervention groups. Descriptive data will include an evaluation of eligibility, recruitment, retention rates and adherence to the intervention, with 95% confidence intervals. Successful adherence to the intervention will be defined as having completed 12 weeks of the computerised cognitive training for a minimum of three, 30-min sessions per week. This is a feasibility study; thus, an estimation of retention rates may be difficult with such as small sample size. Therefore, it is suggested that if retention rates are greater than 75%, we will consider this intervention to be feasible. If the proportion retained is less than this but greater than 65%, we will consider adjusting the intervention to increase this in future investigations.

#### MRI analysis

Potential cognitive training-related changes in the microstructure of white matter pathways of the basal ganglia and motor systems will be assessed with quantitative metrics based on diffusion-weighted (fractional anisotropy, mean and radial diffusivity) and magnetization transfer-weighted MRI (macromolecular proton fraction as proxy MRI metric of axon myelin) [35–37]. Changes in basal ganglia volume will be investigated by extracting subcortical volumes with the FSL-FIRST pipeline [38]. Changes in cerebral blood flow will also be investigated across the whole brain as well as subcortical basal ganglia regions using established methods [39]. The quantitative metrics of tissue structure and blood flow will be correlated with changes in cognition to investigate training-related brain-function associations as well as measures of disease severity, including CAG repeat length, disease burden and Unified Huntington’s Disease Rating Scale (UHDRS) motor scores, using data previously collected and stored as part of the Enroll-HD study.

#### Semi-structured interview analyses

Interview topics will differ slightly depending on allocation and are likely to include how the intervention is perceived/ received, expected or perceived impact on daily routine, expected or perceived impact on family members/carers, overall expectations, expected or perceived acceptability of the intervention, thoughts and views on the randomisation procedure, potential barriers to completing the intervention and general views or comments on cognitive training for people impacted by HD.

Interviews will be transcribed verbatim and analysed using thematic analysis [40]. The data set will be searched to find repeated patterns of meaning, identifying key themes and sub-themes. We will identify contradictory data, as points of contrast as well as similarities in order to understand uptake and engagement with the intervention. Double coding will be utilised to ensure data validity, reliability and integrity. Data will be managed using the qualitative coding software NVivo.

### Monitoring

#### Data monitoring

As this is a non-Clinical Trial of an Investigational Medicinal Product (non-CTIMP) feasibility study which is judged to pose minimal risk to participants, it has been judged that there is no formal need for a DMC. However, the Chief Investigator (CI) will produce regular data monitoring reports to the Centre for Trials Research (CTR) advisory board. The board will assess the data quality and monitor this throughout the study.

#### Harms

The safety of the proposed cognitive training intervention will be monitored throughout the feasibility study by recording of all adverse events (AE). Based on the use of this cognitive intervention programme in a similar patient population with other neurodegenerative diseases [24], we do not anticipate any serious adverse events (SAEs) related to the intervention. However, safety will be monitored throughout and any AEs or SAEs will be recorded using a standard template and reported in line with standard operating procedures and research ethics committee requirements. If, in the opinion of the CI, the SAE was related to the administration of any of the research procedures and was an unexpected occurrence, the CI will report this to the REC within 15 days of becoming aware of the event. If the clinician responsible for the clinical care of participants deems that it is clinically necessary to stop the intervention, the participant will be withdrawn from the intervention. However, the participant will still be invited to complete the outcome assessments. Relapse, worsening of symptoms of HD, death due to HD and hospitalisations for elective treatment of a pre-existing condition will not be reported as SAEs.

#### MRI and incidental findings

MRI scanning at 3 Tesla is a well-established and non-invasive technique for imaging the body and the brain using strong magnetic fields and low-energy radio waves. It does not involve the use of radiation. All persons entering the scanning room are screened for ferromagnetic materials or other factors which cause risk in the presence of a strong magnetic field. The scanner creates significant noise during the scan, which is minimised using earplugs. Verbal contact will be maintained between the MRI operators and the participant through the use of intercom, and a call button will be given to the participant to enable them to stop the scanning procedure at any time. A few people have reported minor side effects during MRI scanning including dizziness, mild nausea, a metallic taste in the mouth, peripheral nerve stimulation and the sensation of seeing flashing lights. These side effects, if experienced, resolve after leaving the magnet, and participants will be informed in the participant information sheet (PIS) of the MRI scanning session of these rare side effects. Some people find being inside an MR scanner claustrophobic; thus, in addition to the 3 Tesla MR machine, CUBRIC also has a ‘mock’ scanner that reproduces, as far as possible, the look and feel of the real MR machine, but without the presence of a magnetic field. Participants will be given the opportunity to lie in this ‘mock’ scanner to acclimatise in advance of the real MRI scan session. If the participant finds the experience in either scanner unpleasant, then he/she would be removed from the scanner.

In some circumstances, changes in the magnetic field within the MRI scanner could make an electric current flow through some of the volunteer’s body, causing peripheral nerve stimulation; this is very rare for the 3 Tesla MRI scanner and not harmful but may be uncomfortable. If two parts of the volunteer’s body were touching (for instance legs crossed), then in very rare occasions, it is possible that an induced current may cause skin heating. Volunteers are instructed to lie with their arms to their sides and legs uncrossed, which stops this from happening. Participants will be informed that CUBRIC is a research centre and therefore cannot provide clinical diagnoses; acquired images of the brain are used for specific research purposes only and not suitable for diagnosis of pathology. However, although the pictures are not diagnostic scans, in the unlikely event of a structural abnormality being noted incidentally, a neuro-radiologist would be asked to review the scan and the clinical lead of the study will be informed. The participant’s general practitioner (GP) will then be notified by the neurologist. There may be a need for the GP to arrange for a formal diagnostic scan.

#### Auditing

The study is subject to inspection by The Jacque and Gloria Gossweiler Foundation and Health and Care Research Wales as the funding organisations. The study may also be subject to inspection and audit by Cardiff University under their remit as sponsor.

#### Consent

Potential participants will be given as long as they need to read the PIS, consider the study and discuss it with the researchers or friends or family, as required. Informed consent will then be obtained by means of a participant dated signature and a dated signature of the person who obtained the informed consent. Participants will be asked during the consent process if their family member, friend or carer can be asked their opinions of cognitive training interventions. If participant consent is provided to ask family members, friends or carers for their opinions, they will be asked to read a separate participant information sheet (PIS) and provide their consent by means of a dated signature and a dated signature of the person who obtained the informed consent. The friend or family member will then be asked to complete a semi-structured interview similar to that described above.

#### Confidentiality

All participant identification and referral procedures as well as procedures for data storage, processing and management will comply with the Data Protection Act 1998. Data will be kept for 15 years in line with Cardiff University’s Research Governance Framework Regulations for clinical research. This data will be stored confidentially on password protected servers maintained on the Cardiff University Network. Data collected on paper forms will be stored in locked cabinets, and electronic data will be stored on secured computer hard drives (password protected) in a swipe card access-controlled building, in compliance with the guidelines set by the Cardiff University Research Governance Framework. The confidentiality of participants will be preserved in accordance with the Data Protection Act 1998. All participants will be allocated a unique study number identifier, and in the case of family members, friends or carers, they will be allocated a unique study number in relation to the participant. All data collected will be held in a linked anonymised form.

#### Dissemination policy

The research team are committed to disseminating the research findings to the general public and to patient groups; therefore, we will seek to present the results at patient open days, engagement events and outreach activities. Furthermore, in order to communicate the research widely, the results may be disseminated via social media, through newsletters and other patient engagement outlets in appropriate language and format for the general public to understand. If a participant indicates that they would like to be informed of the results of the study, a report of the findings will be sent to them at study closure. Where results are presented, they will always be presented in such a way that data from individual participants cannot be identified. In addition to the significant public and patient outreach dissemination, the research findings will be written up for publication in a scientific journal. The results may also be presented at scientific meetings, academic institutions or as part of public engagement and outreach events.

#### Protocol version and amendments

This publication is based on protocol version 4.0 _31.07.2017. The protocol has been written in line with both the CONSORT (Consolidated Standards of Reporting Trials) statement [41] and SPIRIT (Standard Protocol Items: Recommendations for Interventional Trials) guidelines [42]. Any updated protocols will be communicated to the study team via email and telephone as appropriate.

#### Sponsor information

Sponsorship is provided by Cardiff University (SPON 1535-16).

The study receives advisory support from The Centre for Trials Research, Cardiff University.

## Discussion

The cognitive training intervention described in this protocol was developed based on the recommendations of the software providers (HappyNeuron) as well as considering the views of people affected by HD, their family members and friends. During this study, as well as capturing participant views in qualitative semi-structured interviews, we will also aim to consider the views of family members, friends and carers in our analysis of feasibility. Family member, friend and carer involvement in the study will be crucial in determining if this intervention is feasible and able to be completed independently. This will be vital in future studies which may seek to use computerised cognitive training that is completed in participant homes.

In this feasibility study, the proposed computerised cognitive training intervention is supported by home visits and email or telephone reminders. As a result of this, any changes in outcome measures may be attributed to social interaction. Therefore, the control participants will also receive home visits, to control for increased social interaction. Furthermore, it is possible that any observed changes may be attributed to the increased use of a computer. Whilst we have not included them in this in this study, should feasibility be established in future trials, it may be prudent to include additional control groups to explore the generalised effect of computer use in this patient population. Additional control groups could also be considered to explore the effect of home visits, reminders and support provided to participants.

The primary aim of this study is to determine the feasibility of conducting a randomised controlled, computerised cognitive training study for people with HD. Therefore, we will explore the willingness of eligible participants to be randomised, to receive and participate in the intervention, any potential barriers to recruitment or completion and response rates and adherence to the cognitive training programme. This feasibility study has not been powered for hypotheses testing, but the data gathered will inform the design and delivery of future larger trials, including providing vital information to inform sample size estimates. Crucial inferences will be made which will allow an estimation of parameters that can inform definitive and future trials in this specific patient population.

## Abbreviations

AE: Adverse event
CF: Consent form
CI: Chief Investigator
CONSORT: Consolidated Standards of Reporting Trials
CRF: Case report forms
CTR: Centre for Trials Research
CUBRIC: Cardiff University Brain Research Imaging Centre
fMRI: Functional magnetic resonance imaging
GP: General practitioner
HADS: Hospital Anxiety and Depression Scale
HD: Huntington’s disease
IC: Informed consent
IPAQ: International Physical Activity Questionnaire
MBT: Moneybox test
MRI: Magnetic resonance imaging
non-CTIMP: non-Clinical Trial of an Investigational Medicinal Product
PIS: Participant information sheet
QTUG: Quantitative timed up and go
REC: Research ethics committee
SAE: Serious adverse event
SPIRIT: Standard Protocol Items: Recommendations for Interventional Trials
TUG: Timed up and go
UHDRS: Unified Huntington’s Disease Rating Scale
